# The effects of home-based primary care on Medicare costs at Spectrum Health/Priority Health (Grand Rapids, MI, USA) from 2012-present: a matched cohort study

**DOI:** 10.1186/s12913-018-2965-5

**Published:** 2018-03-07

**Authors:** Stephen A. Stanhope, Mary C. Cooley, Linda F. Ellington, Gregory P. Gadbois, Andrew L. Richardson, Timothy C. Zeddes, Jay P. LaBine

**Affiliations:** 1 Analytics and Improvement Department, Spectrum Health (Priority Health), Grand Rapids, MI USA; 2 Medical Executive Department, Spectrum Health (Priority Health), Grand Rapids, MI USA; 3 System Analytics and Data Governance, Spectrum Health, Grand Rapids, MI USA

**Keywords:** Home based primary care, Medical costs, Survival

## Abstract

**Background:**

In the United States, home-based primary care (HBPC) is increasingly proposed as a means of enabling frail elders to remain at home for as long as possible, while still receiving needed medical care. However, there are relatively few studies of either the medical outcome effects or cost benefits of HBPC. In this paper, we examine medical cost and mortality outcomes for enrollees in the HBPC program offered by Spectrum Health/Priority Health (SH/PH), a not-for-profit integrated health care/health insurance system located in Grand Rapids, MI, USA.

**Methods:**

We perform a concurrent matched cohort study. SH/PH HBPC enrollees during 2012–2014 are matched for prior costs, age, sex and comorbidities against controls selected from unenrolled insurance plan members. Twelve and twenty four-month medical costs are compared between HBPC participants and matched controls, overall and conditional on mortality status. Mortality rates of HBPC participants are studied on their own and in comparison to controls.

**Results:**

At 12 and 24 months, in comparison to matched controls HBPC participants show higher ($2933) and lower ($8620) costs respectively. Relative costs and savings of HBPC participants are a function of short term increased costs upon entry into the program (enrollees who survive the first year cost $5866 more than controls); substantial savings at end-of-life (approximately $37,037 in savings relative to controls are realized); and the overall mortality of HBPC participants (mean residual lifespan is 37.75 months from the time of enrollment). We project the present value of lifetime medical cost savings due to enrollment in the HBPC program to be at least $14,336.

**Conclusions:**

The SH/PC HBPC program reduces healthcare costs while enabling frail elders to remain at home. Reduction in costs is obtained at end-of-life and is offset with a smaller initial increase in costs upon enrollment.

## Background

From the perspective of an integrated health care system, achieving the Triple Aim [1] of improved experience of care, improved health and reduced medical costs for a full population of members is a difficult task, requiring specific care plans for particular subpopulations and medical events. Amongst the most difficult subpopulations are medically and functionally compromised elders, who typically have multiple chronic conditions with commensurately high utilization of emergency department (ED) services and hospitalization; may have difficulty obtaining care through normal channels; and may be approaching end of life but are not yet candidates for palliative care. For such individuals, Home Based Primary Care (HBPC) programs have been proposed as a means of assuring the delivery of ongoing medical care in a cost effective manner [2, 3]. However, no strong consensus has been reached regarding either best practices to providing HBPC, or the magnitude of improvements in medical outcomes and costs that might be achieved by doing so [4]. And so, it remains unclear how to best target at risk elders for HBPC, and what services to provide elders enrolled in HBPC programs.

In part, this lack of consensus may be due to the relatively few formal studies of the effects of particular HBPC programs on outcomes and costs, which may lead to the perception amongst payers that HBPC is not worth investing in [5, 6]. In one early study of the Veterans Affairs Home-based Primary Care program [7], it was suggested in a comparison of pre- and post-enrollment utilization patterns that enrollment in HBPC reduced the yearly rate of hospital and ED admissions by 84% and 48% respectively, with commensurate medical cost savings of approximately $10,000 per enrollee. Other similar studies [8, 9] suggested a reduction in hospital admissions of 25% and total costs per patient year of $6148 due to HBPC participation. Stall, Nowaczynski and Sinha [10] provide an overview of a variety of early (pre-2014) observational studies of HBPC programs, concluding that most identify similar reductions in utilization for enrollees, with mixed results for medical cost savings.

More recent studies of HBPC programs have relied on formal quasi-experimental (if not randomized control) study designs to avoid potential selection bias and covariate confounding issues often encountered in pre-post comparisons [11]. Marek et al. [12] suggests in a risk-adjusted analysis of results from a randomized control study that a HBPC program centered around medication self-management saved $447 per month in total medical costs; De Jonge et al. [13] report cost savings of $8477 over 2 years in a matched cohort study of elders enrolled in a HBPC program located in Washington DC; Edwards et al. [14] associate HBPC with a 5.8% reduction in one-year hospitalization rates in an instrumental variables analysis of a home care program operated by the US Department of Veterans Affairs; and Mattke et al. [15] suggest in a differences-in-differences analysis of United Health Group’s HouseCalls program offered to plan members in Arkansas, Georgia, Missouri, South Carolina and Texas that HBPC participants reduce one-year hospital admission rates by 1–14%.

In this paper, we both add to and expand what is understood about the medical service utilization, medical cost, and mortality performance of individual HBPC programs by examining the performance of the statewide HBPC program implemented by the Spectrum Health/Priority Health (SH/PH) integrated healthcare system in October 2012 [16]. This program follows the comprehensive primary care model described by the Agency for Healthcare Research and Quality [4] and has as its objective to deliver comprehensive care to high cost (> $25,000 in both the year prior to enrollment and predicted for the next year) patients with five or more chronic conditions who have an inability to secure care in a primary care physician’s office. We begin our analysis by substantially replicating much of the analysis in De Jonge et al. for our population – we match enrollees 1:1 with controls based on a subset of the criteria used by De Jonge et al., and we compare medical costs and mortality rates of HBPC enrollees with matched controls at 24 months as do De Jonge et al. And, we show outcomes analogous to those reported by De Jonge et al. – a reduction of $8620 in two-year medical costs due to participation in HBPC, with similar mortality rates between enrollees and matched controls.

Our replication of the methods and results obtained by De Jonge et al. serves two purposes - it not only strongly bases our study in context of established methods for studying HBPC programs, but also reinforces the results that have been previously obtained and therefore potentially helps to better establish the intervention itself. We continue our study by expanding upon what was reported by De Jonge et al. We report cost savings at 12 months, incrementally between 12 and 24 months overall and conditional on survival status. These examinations of the data suggest that savings in the SH/PC HBPC program is primarily driven by savings at end of life, and may be offset by initially higher costs. We then estimate a parametric survival function for HBPC participants, and combine it with our conditional estimates of savings to compute a lower bound on the expected present value of lifetime medical cost savings for new program enrollees. Our estimated lower bound of the expected present value of medical cost savings for a new program enrollee is $14,336.

## Methods

### Overview

All enrollees in the SH/PH HBPC program between September 2012 and September 2014 were subjected to 1:1 covariate matching based on their characteristics at the date of enrollment against a larger population observed at times of physician visits. For each case and potential control, medical claims data was used to calculate prior enrollment medical costs, utilization and comorbidities, which were used as the basis of matching. Post-enrollment, medical claims data was used to calculate medical costs and state records were used to determine mortality status. Medical cost and mortality rates were compared between cases and controls.

### Setting, participants, intervention

Spectrum Health/Priority Health is a non-profit integrated health care system providing health care services (SH) and insurance (PH) to residents of Michigan, USA. On the provision side, Spectrum Health is comprised of 12 hospitals and 181 ambulatory and service sites in a 13 county service area, primarily in Western Michigan. Priority Health, a member of the Association of Community Health Plans, provides comprehensive health insurance coverage to over 650,000 members in the individual, commercial, Medicaid and Medicare markets, with the majority residing in Western Michigan (Kent, Ottawa, and Allegan counties).

As of second quarter 2015, approximately 422 patients served by SH with health insurance coverage through PH have been enrolled in the HBPC program. The majority of these patients are located in Western Michigan. Prospective members for the program are identified and referred by the PH Care Management department, hospitals, and primary care physicians located within and outside of the Spectrum Health System network. Initial screening is performed to verify eligibility (patients are eligible if they have at least five chronic conditions and meet medical cost criteria), and identify possible safety concerns and compliance issues including documented mental health conditions, housing, substance abuse and safety concerns. Patients that meet eligibility criteria are enrolled upon written consent.

### Intervention

The SH/PH HBPC model delivers comprehensive, patient-centered care to the home on a regular or as-needed basis to provide intensive outpatient management of chronic conditions through monitoring and evaluation, therapy, pharmacy, social work, and ancillary services. Additionally, the patient is engaged in advanced care planning and Hospice care discussion, and there is effort made to assure that the patient’s desired course of care at end of life are followed.

The core team of care is comprised of a primary care physician, nurse practitioner or physician assistant, and nurse care manager. Upon initial enrollment, the nurse care manager initiates contact with the patient and a family member/caregiver. An appointment is scheduled to meet, obtain a consent form, and take medical history. Following enrollment, patients meet on average every 4 to 6 months with the primary care physician, every 2 to 3 months with the physician assistant or nurse practitioner, and once a month with the nurse care manager. Scheduled visitations are frequently modified to meet the individual patient’s needs for actual and/or ancillary services.

### Study participant selection

Participants in the SH/PH HBPC program and potential controls were observed between September 2012 and September 2014. Potential controls were observed at the time of a physician visit. There were 253 enrollees and 280,134 potential controls.

### Outcomes

Primary outcomes are allowed medical costs (costs charged to PH by providers, inclusive of inpatient and outpatient facility costs; professional services costs; and pharmacy costs) and mortality. Outcomes were measured at 12 and 24 months from HBPC enrollment. All cases and matched controls were included in follow up analysis; medical claims data was used to determine costs, and state records were used to determine mortality. No time discounting was performed on medical costs.

### Statistical analysis

Comparisons of costs and mortality rates were made between HBPC participants and controls matched 1:1 on the basis of total medical costs 4 months prior to enrollment (within 25%); prior in-patient and skilled nursing facility (SNF) utilization 4 months prior to enrollment (dichotomous, exactly matched); age (within 10 years); Alzheimer’s disease or chronic mental illness status (dichotomous, exactly matched); coronary heart disease status (dichotomous, exactly matched); cerebrovascular disease status (dichotomous, exactly matched); diabetes status (dichotomous, exactly matched); chronic obstructive pulmonary disease status (dichotomous, exactly matched). Such matching was performed for the full population, and conditional on mortality status. Note that these are the same criteria used by De Jonge et al. with exception of the omission of sex and arthritis status, which did not change the outcome of the study but did reduce the number of successful matches. In the event of more than one potential match for a given HBPC participant, the match was assigned to be the control with closest prior medical costs. If no match could be made for a participant, it was rejected from further analysis.

After matching, average total medical, pharmaceutical (RX), in-patient (IP) and out-patient (OP) costs and mortality rates at 12 and 24 months were compared between the HBPC group and matched controls (overall and conditional on mortality status). In the event that a full observation period for a subject was not completed, costs were imputed [17]. For long term (> 13 months) HBPC participants who had not reached end of life, we compared average monthly costs to matched controls in the initial 12 months, months 13–24, and months 1–24 of enrollment. For all cost comparisons, average differences favoring HBPC participants were taken to be indicative of cost savings. Significance testing for cost savings was performed via standard t-tests against the null of no difference, and testing for differences in mortality rates was done with Fisher exact tests (again against the null of no difference). Confidence intervals (at the 95% level) for cost savings were obtained from the t-test. We estimated a parametric (Weibull) survival function for the full HBPC population, and used it to compute the mean residual lifespan for a HBPC member from the time of enrollment, as well as 12 and 24 month survival probabilities. The survival probabilities were used in conjunction with 12 and 24 month estimates of costs and savings conditional on mortality and an assumed 1% discount rate to calculate a present value estimate of the savings due to enrolling a member in the HBPC program.

## Results

Post-matching, 210 HBPC participants were compared with 210 controls. Tables 1 and 2 provide baseline characteristics and follow-up period medical costs for the participant and control groups respectively. With respect to baseline characteristics, those which were explicitly matched upon are balanced across groups, and those which were not show good balance. During the follow-up period, at 12 months HBPC participants showed increased costs relative to matched controls ($2933, or $244 per month). However, at 24 months HBPC participants show savings of $8620 ($359 per month) relative to controls. At both time horizons, HBPC participants had proportionally lower and greater in- and outpatient expenses than controls respectively.

**Table 1:** Treatment and matched control characteristics

Characteristic	Treats. (*n* = 210)	Controls (n = 210)
% Female	61	54
Median age	79	77
Mean 4 month pre-enrollment total medical cost	$13,075	$13,001
Mean 4 month pre-enrollment RX cost	$1452	$1484
Mean 4 month pre-enrollment IP cost	$6200	$6955
Mean 4 month pre-enrollment OP cost	$2701	$1895
% with 4 month pre-enrollment IP stays	40.0	40.0
% with 4 month pre-enrollment SNF stays	14.8	14.8
% with Alzheimer’s disease or chronic mental illness	36.2	36.2
% with coronary heart disease	68.6	68.6
% with cerebrovascular disease	33.3	33.3
% with congestive heart failure	31.4	31.4
% with diabetes	45.2	45.2
% with chronic obstructive pulmonary disease	33.8	33.8
% with arthritis	28.1	27.6

Table 1 provides demographic, medical cost and utilization, and morbidity patterns of the matched HBPC participant and control samples. The samples demonstrate balance for covariates explicitly matched upon (age; total medical cost; Alzheimer’s disease or CMI; CHD; CVD; CHF; diabetes; COPD) as well as those not (sex; RX, IP and OP costs; arthritis)

**Table 2:** Medicare allowed costs during follow-up – overall HBPC participants/matched controls

		*N*	Total cost	RX cost (% total)	IP cost (% total)	OP cost (% total)	Savings, 95% CI, *p*-value
12 months	HBPC	210	$28,621	$4937 (17.24%)	$9783 (34.18%)	$7462 (26.07%)	($2933) ($7672), $1806 *p* = 0.225
	Ctrl	210	$25,688	$4677 (18.21%)	$10,573 (41.15%)	$4576 (17.81%)	
24 months	HBPC	210	$39,738	$6798 (17.11%)	$12,558 (31.60%)	$11,003 (27.69%)	$8620 $1959, $15,281 *p* = 0.011
	Ctrl	210	$48,358	$8600 (17.78%)	$19,859 (41.06%)	$8422 (17.41%)	

Table 2 provides patterns of medical costs and savings for the matched HBPC participant and control samples at 12 and 24 months. HBPC participants show greater expenditure than matched controls at 12 months. At 24 months, this has reversed, and HBPC participants show medical cost savings driven primarily by reductions in in-patient costs

To understand the difference in 12 and 24 month medical cost savings due to participation in HBPC, we provide differences in medical costs between participants and matched controls conditional on mortality in Table 3 and differences in medical costs over time for long term HBPC participants in Table 4. In Table 3, it can be observed that HBPC members show substantial cost advantages at end-of-life relative to matched controls at either the 12 or 24 month time horizons. Such savings can be attributed to reductions in in-patient costs and are offset by increased costs prior to end of life, especially in the first 12 months of enrollment (Table 3, 12 months and Table 4). Mortality rates are provided in Table 5. The HBPC population shows slightly but statistically insignificantly higher mortality rates than controls.

**Table 3:** Medicare allowed costs during follow-up – participants and controls matched for mortality

			*N*	Total cost	RX cost (% total)	IP cost (% total)	OP cost (% total)	Savings, 95% CI, *p*-value
12 months	*Alive*	HBPC	176	$28,373	$5394 (19.01%)	$9272 (32.68%)	$7041 (24.82%)	($5866) ($10,606), ($1125) *p* = 0.015
		Ctrl	176	$22,507	$4833 (21.69%)	$8358 (37.13%)	$3960 (17.59%)	
	*Dead*	HBPC	20	$21,273	$1847 (8.68%)	$10,138 (47.66%)	$5373 (25.25%)	$37,037 $11,552, $62,522 *p* = 0.006
		Ctrl	20	$58,310	$4174 (7.16%)	$38,119 (65.37%)	$4253 (7.29%)	
24 months	*Alive*	HBPC	161	$41,716	$6219 (14.91%)	$13,381 (32.08%)	$12,398 (29.72%)	$726 ($6464), $7916 *p* = 0.84
		Ctrl	161	$42,442	$8832 (20.81%)	$14,193 (33.44%)	$8815 (20.77%)	
	*Dead*	HBPC	31	$28,116	$2154 (7.66%)	$12,516 (44.51%)	$7907 (28.12%)	$27,610 $8077, $47,142 *p* = 0.007
		Ctrl	31	$55,726	$4214 (7.56%)	$30,732 (55.14%)	$8800 (15.79%)	

Table 3 provides patterns of medical costs and savings for matched (conditional on mortality status) HBPC participant and control samples at 12 and 24 months. HBPC participants show substantial savings at end of life relative to controls, driven strongly by reductions in in-patient costs. Prior to end of life, HBPC participants are more expensive initially, and cost neutral after the first 12 months of enrollment

**Table 4:** Average monthly costs of long-term HBPC participants and matched controls prior to end of life

	N	Months 1–12	Months 13–24	Months 1–24
HBPC	58	$2278	$2428	$2367
Control	58	$1671	$2543	$2106
Difference, p-value		($607) *p* = 0.107	$115 *p* = 0.881	($261) *p* = 0.574

Table 4 examines the monthly medical costs of uncensored (at 24 months), surviving HBPC participants relative to controls. Consistent with Table 3, the medical costs of HBPC participants are initially greater and long term equivalent to controls

**Table 5:** Mortality rates for HBPC cases, matched controls

		Deaths	Mortality rate	Relative risk, *p*-value
12 months	HBPC	31	15%	1.56 *p* = 0.182
	Control	21	10%	
24 months	HBPC	42	20%	1.21
	Control	36	17%	*p* = 0.531

Table 5 provides mortality rates and relative risks of HBPC participants relative to controls. HBPC participants show slightly but insignificantly greater mortality rates, likely due to unmatched criteria used to select HBPC participants

To conclude, we examined the relationship between mortality and cost savings in the HBPC population by first estimating a parametric survival function based on the full population of 253 members, and then using that survival function to obtain a lower bound estimate of the present value of lifetime medical cost savings accruing to HBPC participation. The shape and scale of the estimated survival function were 1.166 and 1180.265 respectively, indicating a mean survival time from the date of enrollment of 37.75 months with 12 and 24 month mortality rates of 22.47% and 21.04% respectively (56.49% of participants are estimated to survive longer than 24 months). Applying these survival probability estimates to the cost savings results provided in Table 3 (a $37,037 and $27,610 cost advantage to participants dying within 12 and 24 months of enrollment, and a $726 cost advantage to participants surviving longer than 24 months), we compute a present value of $14,336 in medical cost savings to HBPC participants over 24 months (assuming a 1% discount rate). Assuming that cost advantages at death will persist beyond 24 months, and that any further cost disadvantages for survivorship will not outweigh such advantages, $14,336 would be the lower bound on the present value of lifetime medical cost savings.

## Discussion

In this paper, we examined medical cost and mortality outcomes for enrollees in a home based primary care (HBPC) program at the Spectrum Health/Priority Health (SH/PH) integrated healthcare system in Michigan. To do so, we followed and built on much of the analysis described in De Jonge et al. (2014). We obtained a matched cohort and showed that the matched set of SH/PH HBPC enrollees and controls are balanced with respect to the same criteria used by De Jonge et al. We examined differences in medical costs between HBPC members and matched controls at 12 and 24 months, both as a full group and conditional on mortality status, and compared the medical costs of long term HBPC enrollees with controls. Last, we examined the mortality of HBPC enrollees, both in comparison to controls and on its own, and computed a present value of total medical cost savings over the residual lifetime of a patient once enrolled.

Because we follow De Jonge’s analysis closely, it is useful to compare results across studies. Both studies show a statistically significant reduction in medical costs at the 24 month time horizon (De Jonge $8477 per person in comparison with $8620 here). Both show that these savings are due to reductions in in-patient care, and similar arguments are made with respect to which participants show savings: In De Jonge’s study savings are primarily observed in participants with high frailty, and here savings are observed in participants at end-of-life. With respect to mortality, in neither study is a significant increase observed in HBPC participants. We add to De Jonge’s analysis by showing that, in the SH/PH HBPC program savings obtained at end-of-life are offset by increased costs in the initial year of the program, by computing a survival function for HBPC participants, and estimating a lower bound on the present value of the total lifetime medical cost savings per enrolled member ($14,336). Our observation that new HBPC members are likely to show increased medical costs in the short term is consistent with those made by Kaye et al. [18] in a study of statewide expenditures post expansion of Medicare funded home and community based services. Our projected lower bound on the present value of lifetime savings includes these short term increased medical costs, and but not any possible savings (or costs) accruing at death or upon survival past 2 years. We believe that there ought to be continued cost savings accruing at death as well as to survivors, and that the present value of lifetime savings due to HBPC participation will therefore be greater than this lower bound.

In common with the results demonstrated by De Jonge et al., our results have limitations: They are not the results of a randomized study; we do not have estimates of disease severity for either our treatment or control populations; and it is unclear to what extent the population under study is representative of the entire nation’s elderly population. However, many of the strengths of De Jonge’s study are also represented here – in particular, the utilization of longitudinal claims data not subject to recall bias, and a quasi-experimental design based on covariate matching.

Given these similarities, there are some differences. De Jonge et al. make use of the JEN Frailty Index as a matching criterion, whereas we do not (and in fact had no access to this metric). We note that the JEN Frailty Index is computed based on administrative claims data, and we suppose that there would be reasonable correlation between it and the matching criteria used here. In De Jonge’s study, 5 year age bands are used for matching, whereas we use 10 to increase the number of matched HBPC participants successfully matched to a control. The results described here are not substantially impacted by this change. For example, if we restrict our matching to a 5 year age band and examine mortality matched cost differences between HBPC participants and controls, we obtain a $5088 cost disadvantage at 12 months for surviving participants (*p* = 0.029) and a $27,906 savings for participants who die (*p* = 0.074). At 24 months, surviving participants show a $1533 cost disadvantage (*p* = 0.616) and those who die a $24,248 cost savings (*p* = 0.047).

With respect to other issues either particular to this study or shared between our study and De Jonge’s, we note the following: 1) The definition of costs used here is based on provider charges to PH. Typically, not all charges are paid (for example, due to copays and deductibles), and if we were to have based our study on paid amounts we would expect to see a reduction in the amount of cost savings as stated. We believe that provider charges provide the best representation of actual costs incurred. 2) We note that other statistical methodologies, such as those based on the propensity score, might serve as the basis of reasonable analyses of our data. In general, we prefer direct covariate matching when the pool of putative controls is large enough to successfully match a large proportion of program enrollees. In our study, we match approximately 90% of program participants with a control.

## Conclusions

In total, our results add to the growing body of literature suggesting that providing health care at home in the community environment can reduce medical costs for elderly patients with complex disease burdens and functional impairments. We suggest that, for such care programs, achieving overall cost savings might require balancing increased short-term costs due to increased medical utilization with longer term savings due to advanced care planning. Such balancing may be achieved by selectively targeting prospective enrollees. We continue to monitor the growing SH/PH HBPC population with a particular interest in the quality of patient care and patient/family satisfaction, and are using our current results to improve said program targeting.

## Abbreviations

ED: Emergency department
HBPC: Home-based primary care
IP: In-patient
OP: Outpatient
PH: Priority Health
RX: Prescription
SH: Spectrum Health
SNF: Skilled nursing facility
